# Up-and-coming anti-epileptic effect of aloesone in *Aloe vera*: Evidenced by integrating network pharmacological analysis, *in vitro*, and *in vivo* models

**DOI:** 10.3389/fphar.2022.962223

**Published:** 2022-08-12

**Authors:** Yan Wang, Chang Li, Zhongyv Xiong, Niangen Chen, Xuesong Wang, Junyv Xu, Yuemei Wang, Longfeng Liu, Hang Wu, Caihui Huang, Aiqin Huang, Jiajia Tan, Youbin Li, Qifu Li

**Affiliations:** ^1^ Key Laboratory of Tropical Translational Medicine of Ministry of Education, Hainan Key Laboratory for Research and Development of Tropical Herbs, Department of Neurology, School of Pharmacy, The First Affiliated Hospital, Hainan Medical University, Haikou, China; ^2^ Key Laboratory of Brain Science Research and Transformation in Tropical Environment of Hainan Province, Department of Neurology, The First Affiliated Hospital of Hainan Medical University, Hainan Medical University, Haikou, China; ^3^ College of Veterinary Medicine, Southwestern University, Chongqing, China

**Keywords:** *Aloe vera*, aloesone, network pharmacology, glutamate-induced HT22, PTZ-induced seizure, c-SRC

## Abstract

**Background:** *Aloe vera* is a medically valuable plant with anti-epileptic activity; however, its mechanism of action remains unknown. In this study, network pharmacological, *in vitro*, and *in vivo* experiments were carried out to explore the potential anti-epileptic components and targets of *Aloe vera*.

**Methods:** The main active components of *Aloe vera* were identified by searching the Traditional Chinese Medicine System Pharmacology database. Targets of *Aloe vera* were predicted using SwissTargetPrediction, whereas information about the epilepsy disease targets was obtained from Gene Cards. The protein–protein interaction network and core targets were screened according to the topological structure and CytoNCA plugin. The glutamate-induced HT22 cell line and pentylenetetrazol-induced seizure rats were used to confirm the effect of aloesone by detecting reactive oxygen species (ROS) and apoptosis, and predicting the targets.

**Results:** A total of 14 core active components were selected based on the screening criteria of oral bioavailability ≥30% and drug-likeness ≥ 0.10. Four compounds, namely linoleic acid, aloesone, isoeleutherol glucosiden qt, and anthranol, demonstrated the potential ability of crossing the blood-brain barrier. A total of 153 targets associated with epilepsy were predicted for the four compounds. Moreover, after network analysis with CytoNCA, 10 targets, namely, MAPK1, SRC, MARK3, EGFR, ESR1, PTGS2, PTPN11, JAK2, PPKCA, and FYN, were selected as the core genes, and SRC, which has been predicted to be the target of aloesone and anthranol, exhibited the highest subgraph centrality value. *In vitro* experiments confirmed that aloesone treatment significantly inhibited the glutamate-induced neuronal injury by reducing the intracellular ROS content and the early phase of apoptosis. Additionally, treatment with 50 mg/kg aloesone resulted in anti-seizure effects by reducing the seizure score and prolonging the latent period in acute and chronic rats. Furthermore, aloesone treatment increased the phosphorylation of c-SRC at Y418 and reduced the phosphorylation at Y529, simultaneously activating c-SRC.

**Conclusion:** Integrating network pharmacology with *in vitro* and *in vivo* experiments demonstrated that aloesone, which inhibited seizure by activating c-SRC, is a potential anti-seizure compound present in *Aloe vera*.

## Graphical Abstract

Aloesone from Aloe vera inhibits glutamate-induced HT22 injury and PTZ-induced seizures by increasing the activation of c-SRC, acting as a potential anti-seizure drug.
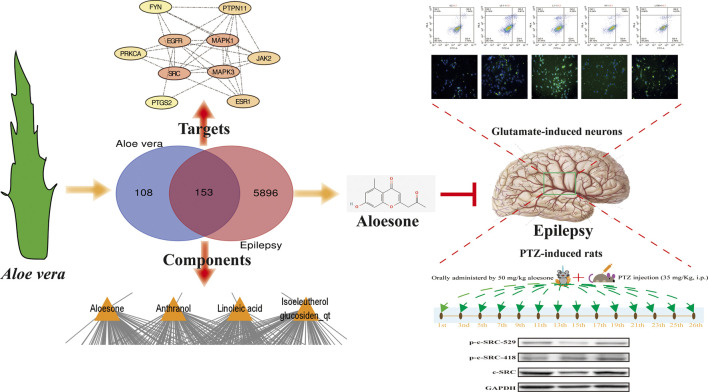

## 1 Introduction

Epilepsy is the most prevalent chronic neurological disorder affecting approximately 70 million people worldwide (Singh and Trevick, 2016). Recurring seizures are associated with comorbidities such as anxiety, mood disorders, and hypertension and result in a financial burden (Quintana et al., 2021).

The use of anti-epileptic drugs (AEDs) is currently the main treatment strategy for epilepsy, and most AEDs target the regulation of voltage-gated channels (e.g., phenytoin or carbamazepine for sodium, ethosuximide for calcium, and retigabine for potassium channels), augment inhibitory neurotransmission through gamma-aminobutyric acid type A receptors (e.g., benzodiazepines and tiagabine), reduce excitatory neurotransmission through glutamate receptors (e.g., perampanel), or regulate neurotransmitter release through presynaptic terminals (e.g., levetiracetam and gabapentin). However, one-third of patients with epilepsy are refractory to AEDs despite following appropriate treatment protocols (Voll et al., 2015). Although studies have been conducted to discover new AEDs, such as vigabatrin and cannabidiol, most of them only focused on model efficacy and reduced adverse effects. Until now, refractory epilepsy remains prevalent (Iannone et al., 2021; Janson and Bainbridge, 2021). Hence, it is necessary to uncover new AEDs that can better control seizures and minimize adverse effects.

*Aloe vera* has been traditionally used in several countries, including Greece, Egypt, India, and China (Surjushe et al., 2008). It contains various compounds, including anthraquinones, anthrones, chromones, alkaloids, and flavonoids (Kumar et al., 2019), which exhibit anti-tyrosinase (Mikayoulou et al., 2021), anti-cancer (Majumder et al., 2020; Cheng et al., 2021), and anti-diabetic effects (Prasannaraja et al., 2020). A previous study showed that complex N-095 containing 10 mg of aloe wood decreased the frequency of tonic seizures induced by pentylenetetrazol (PTZ) and prolonged the survival time (Inoue et al., 2005). Other studies have demonstrated that an aqueous extract obtained from the leaves of *Aloe vera* could approximately double the latency to onset period of clonic convulsions and decrease their duration by 300% in PTZ-induced acute rats (Rathor et al., 2014), confirming the anti-epileptic effect of *Aloe vera*. However, the specific compounds that exhibit these anti-seizure effects are unclear. Although *Aloe vera* is a non-toxic plant, its topical or oral use causes adverse effects, including intraoperative bleeding, acute oliguric renal failure, and hepatotoxicity in humans (Luyckx et al., 2002; Lee et al., 2004; Guo et al., 2010; Guo and Mei, 2016). Therefore, identifying the specific anti-seizure compound in *Aloe vera* is vital for its utilization.

In this study, network pharmacology was applied to explore the anti-seizure compounds in *Aloe vera*, and *in vitro* and *in vivo* experiments were performed on neuronal cell lines and rats to confirm the anti-epileptic effects.

## 2 Methods

### 2.1 Network pharmacological analysis

#### 2.1.1 Identifying the main active ingredients of *Aloe vera* and predicting targets of the main compounds

The traditional Chinese medicine system pharmacology database and analysis platform (TCMSP, https://old.tcmsp-e.com/tcmsp.php) was used to identify the active ingredients of *Aloe vera*. Ingredients whose toxicokinetic absorption, distribution, metabolism, and excretion (ADME) met the criteria (oral bioavailability (OB) ≥ 30%, drug-likeness (DL) ≥ 0.10) were considered the main compounds. SwissADME (http://www.swissadme.ch/) was used to predict the potential of the ingredients to cross the blood brain barrier (BBB). The related compounds were then input into PubChem (https://pubchem.ncbi.nlm.nih.gov/) to obtain their molecular structures, while the canonical simplified molecular input line entry system (SMILES) was used for target identification on different websites, including the similarity ensemble approach (https://sea.bkslab.org/) and SwissTargetPrediction (https://www.swisstargetprediction.ch/).

#### 2.1.2 Construction of an “herbs-components-targets” network and analysis of gene functions

Information about the target genes for epilepsy was collected from GeneCards (https://www.genecards.org/, version 4.9.0), a database integrated with genome, transcriptome, proteome, and genetics, as well as clinical and functional information from 150 web sources. Overlapping genes between the core active ingredient target of *Aloe vera* and the disease targets of epilepsy were retrieved using the Venn diagram (https://bioinformatics.psb.ugent.be/webtools/Venn/). Geno Ontology (GO) gene functions and biochemical pathways of overlapping genes were enriched using the web-based annotation tool DAVID v6.8 (https://david.ncifcrf.gov/tools.jsp), providing GO terms in the categories of biological process (BP), cellular component (CC), molecular function (MF), and Kyoto Encyclopedia of Genes and Genomes pathways (KEGG). *p* < 0.05 was set as the significance threshold.

#### 2.1.3 Screening of core genes

The protein-protein interaction (PPI) network of the overlapping genes with high confidence was obtained using the STRING online tool (https://string-db.org/). Core genes were analyzed using CytoNCA in Cytoscape 3.9.1 (https://www.cytoscape.org/). The three most important parameters selected to screen core composite targets of Indigo were degree centrality (DC), closeness centrality, and betweenness centrality (BC) (Tang et al., 2015). According to relevant reports, targets with BC and CC values higher than those of the median as well as a DC value twice that of the median were selected to obtain more accurate core targets (Pan et al., 2022).

#### 2.1.4 Molecular docking

The crystallographic structure of the c-SRC protein was downloaded from the Protein Data Bank (No. 3F3V) and generated using the protein preparation wizard and receptor grid generation modules. Simultaneously, preparation mode of aloesone was initially set by the LigPrep module with ionization generated states at target pH = 7.0 ± 2.0. OPSL3 was selected in the s. Force field option, while other parameters were set to their default values. Then, the ligand docking module with standard settings for standard and extra precision was used to dock aloesone and c-SRC. The docking-structure figure was presented using the PyMOL molecular graphic system (Xu et al., 2020).

### 2.2 *In vitro* experiment

#### 2.2.1 Cell culture

The murine hippocampal cell line HT22 was purchased from BNCC (Bena Culture Collection, Beijing, China) and grown in high glucose Dulbecco’s modified eagle medium (Gibco, California, United States), supplemented with 10% fetal bovine serum (CLARK, Virginia, United States) and streptomycin/penicillin (Biosharp, Anhui, China) in a humidified incubator with 5% CO_2_ at 37°C.

#### 2.2.2 Cell viability

Cell viability was analyzed using a Cell Counting Kit-8 (CCK8, Biosharp). Briefly, the cells were seeded and cultured in 96-well microplates at a density of 1×10^5^/ml. The cells were then treated with various concentrations (0.1, 1, 10, 100, and 1,000 µM) of aloesone (synthesized according to a previous study (Kim et al., 2017)) for 24 h and incubated with 20 µL CCK8 reagent for 30 min. Absorbance was measured at 450 nm with a microplate reader (Spectra MAX 190, Molecular Devices, San Jose, United States) using wells without cells as blanks. Cell proliferation was evaluated by measuring the absorbance according to a previously described method (Wang et al., 2021).

#### 2.2.3 Measurement of reactive oxygen species generation

The cells were exposed to glutamate (5 mM, Sigma, Saint Louis, Missouri, United States) and aloesone (0.1, 1, 10, and 100 µM) for 24 h. Then, the cells were incubated with 10 µM 2′,7′-dichlorofluorescin diacetate (Sigma) for 30 min. Fluorescent images were obtained using a fluorescent microscope (Zeiss X-Cite, Oberkochen, German), and the fluorescent intensity was used as an indicator of intracellular ROS levels measured using flow cytometry (NovoCyte, Agilent, Palo Alto, United States) with the fluorescein isothiocyanate (FITC) channel.

#### 2.2.4 Hoechst 33258 staining

The chromatin-specific dye, Hoechst 33258, was used to visualize the morphological alterations in the HT22 cells. The cells were fixed with 4% paraformaldehyde for 30 min, followed by washing with phosphate-buffered saline (PBS) three times (Zhang et al., 2020). After incubation with a working solution of Hoechst for 15 min, the cells were observed and photographed using a magnification microscope (Zeiss X-Cite) (Zhang et al., 2020).

#### 2.2.5 Annexin Ⅴ–FITC analysis for apoptosis

HT22 cells treated with glutamate and aloesone were harvested using trypsin and incubated with annexin Ⅴ–FITC and propidine iodide (PI) for at least 5 min (Boster, Wuhan, China). The samples were then examined using a flow cytometer (NovoCyte).

#### 2.2.6 Immunofluorescence staining

First, the cells, at a density of 1×10^5^/ml, were incubated for 30 min in 5% bovine serum albumin to block nonspecific binding, and then incubated overnight at 4°C with mouse or rabbit monoclonal antibodies against proto-oncogene c-SRC (1:50, Santa, Santa Cruz, United States) and p-c-SRC (anti Y418, 1:50, Santa; anti Y529, 1:200, Abcam, Cambridge, United Kingdom). After washing with PBS, the cells were stained with a FITC-conjugated secondary antibody for 2 h at 37°C, followed by 4′,6-diamidino-2-phenylindole (DAPI, Boster) for 5 min in the dark. A magnification microscope (Zeiss X-Cite) was used to observe the stained cells. Then, 10 microscopic fields were randomly viewed at a ×200 magnification (Li et al., 2021). Subsequently, fluorescent images were processed using the ImageJ software (1.52a, Wayne Rasband, United States) to adjust the brightness and contrast. All image acquisition and analyses were conducted by an investigator blinded to the experimental conditions (Dou et al., 2021).

### 2.3 *In vivo* experiment

#### 2.3.1 PTZ-induced acute model

Male Sprague-Dawley rats (250 g, certificate no. SCXK 2014-0011) were obtained from Tianqin Biotechnology Co., Ltd. (Hunan, China) and acclimatized to constant temperature and moisture conditions for 1 week. All efforts were made to reduce the number of animals used and to minimize animal suffering and discomfort.

The rats were divided into three groups (PTZ group, 50 mg/kg of aloesone group, and 100 mg/kg of aloesone group), via oral administration of 20% DMSO, 50 mg/kg, or 100 mg/kg of aloesone, respectively, for 30 min before the intraperitoneal injection of PTZ (60 mg/kg). The seizure score and latent time were observed within 30 min.

#### 2.3.2 PTZ-induced chronic model

To evaluate the effects of aloesone on chronic epilepsy, the rats were divided into four groups, as follows:

1) Sham group: rats orally administered 20% dimethylsulfoxide (DMSO) 30 min before the intraperitoneal injection of 0.9% saline.
2) PTZ group: rats orally administered 20% DMSO 30 min before the intraperitoneal injection of 35 mg/kg of PTZ.
3) Aloesone (50 mg/kg) group: rats orally administered 50 mg/kg of aloesone 30 min before the intraperitoneal injection of 35 mg/kg of PTZ.
4) Valproic acid (VPA, 350 mg/kg) group: rats orally administered 350 mg/kg of VPA 30 min before the intraperitoneal injection of 35 mg/kg of PTZ.

Aloesone was dissolved in 0.9% saline and 20% DMSO. All rats, except those in the sham group, were alternately injected with 35 mg/kg PTZ 14 times in 26 days. The seizure scores and latent times were observed within 30 min of PTZ administration.

#### 2.3.3 Seizure observation

Seizures were scored according to Racine’s criteria, as follows: stage 0, no response; stage 1, chewing and face twitching; stage 2, neck spasms and head nodding; stage 3, unilateral forelimb clonus and twitching; stage 4, rearing with bilateral forelimb clonus; and stage 5, generalized tonic-clonic seizures with loss of postural control (Racine, 1972). Latency to seizure onset was defined as the time elapsed between PTZ injection and the first observed seizure response (Huizenga et al., 2017).

#### 2.3.4 Western blotting

The rat hippocampi were extracted from the brain and homogenized using radio immunoprecipitation assay lysis buffer and a phosphorylation inhibitor. Protein content was determined using a BCA kit. Proteins were electrophoresed on SDS-PAGE gels and transferred onto polyvinylidene fluoride membranes. After blocking with 10% nonfat milk powder, the membranes were incubated with rabbit anti-SRC (1:5,000), anti-p-c-SRC (Y418, 1:500; Y529, 1:5,000), and secondary antibodies.

### 2.4 Statistical analysis

All data are expressed as mean ± standard deviation (SD). Normal distribution analysis was performed using the Shapiro–Wilk test. Data with a normal distribution were analyzed using Student’s t-test for two groups and a one-way analysis of variance (ANOVA) for multiple groups. A repeated two-way ANOVA with Benjamini and Kruskal–Wallis test with Dunn’s were used to analyze differences in the latent period and seizure score, respectively, among the PTZ, aloesone, and VPA groups at different time points. Otherwise, the non-normally distributed data were analyzed using the Kruskal–Wallis test. The results were considered significant at *p* < 0.05. Statistical analysis and figure generation were performed using GraphPad Prism (version 9.0.0).

## 3 Results

### 3.1 Prediction of targets of *Aloe vera* and visualization of the herbs-components-targets network

**FIGURE 1:**
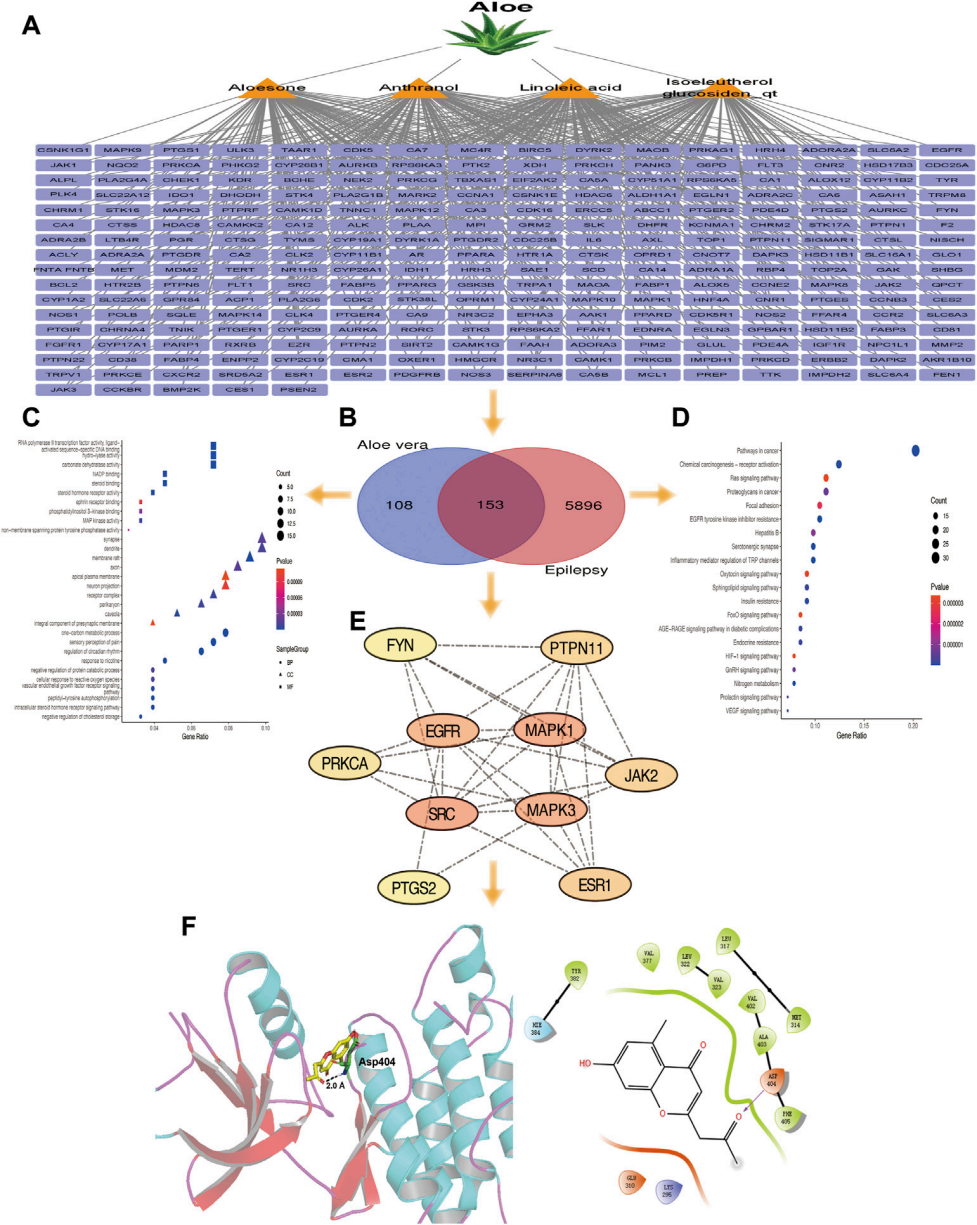
**(A)** The 261 predicted targets of linoleic acid, aloesone, isoeleutherol glucosiden qt, and anthranol. **(B)** A total of 153 targets overlapped between the four compounds and epilepsy. **(C)** GO enrichment of 153 overlapping targets. **(D)** Pathway enrichment analysis of 153 overlapping targets. **(E)** The 10 core targets extracted from the 153 overlapping targets by CytoNCA. Color varying from yellow to red represents the degree value from 16 to 34. **(F)** Binding model of aloesone with active pocket of c-SRC kinase. The aloesone and the key residue are presented by yellow and green sticks, respectively. Hydrogen bonds are shown as black dashes.

A total of 14 active ingredients were obtained from TCMSP, based on OB ≥ 30% and DL ≥ 0.10. Among them, linoleic acid, aloesone, isoeleutherol glucosiden qt, and anthranol showed high potential in crossing the BBB (Table 1). This finding showed that, these four compounds may possess anti-epileptic effects. Using the canonical SMILES number of these four ingredients, 261 potential targets of *Aloe vera* were identified from the similarity ensemble approach and SwissTargetPrediction (Figure 1A). Among them, 153 composite targets overlapped between the targets of indigo chinensis (linoleic acid, aloesone, isoeleutherol glucosiden qt, and anthranol) and epilepsy (Figure 1B).

**TABLE 1:** The 14 active ingredients of *Aloe vera*.

Molecule ID	Molecule name	Molecule Weight	OB (%)	DL	BBB Permeant
MOL000131	Linoleic acid	280.5	41.90	0.14	Yes
MOL001439	Arachidonic acid	304.52	45.57	0.20	No
MOL002773	Beta-carotene	536.96	37.18	0.58	No
MOL000359	Sitosterol	414.79	36.91	0.75	No
MOL000471	Aloe-emodin	270.25	83.38	0.24	No
MOL005040	Aloveroside_qt	232.25	44.58	0.12	No
MOL005043	Campest-5-en-3beta-ol	400.76	37.58	0.71	No
MOL005051	Aloeresin C	702.72	34.99	0.50	No
MOL005054	Aloesone	232.25	59.72	0.12	Yes
MOL005059	Isoeleutherol glucosiden_qt	244.26	39.16	0.17	Yes
MOL005062	Anthranol	194.24	32.38	0.12	Yes
MOL000675	Oleic acid	282.52	33.13	0.14	No
MOL000953	Cholesterol	386.73	37.87	0.68	No
MOL000098	Quercetin	302.25	46.43	0.28	No

### 3.2 GO and KEGG enrichment analysis

GO and pathway enrichment analyses were performed for the 153 targets that were significantly enriched in the BPs of one-carbon metabolic process, sensory perception of pain, and regulation of circadian rhythm. The synapse, dendrite, and membrane raft were enriched in CC. With respect to MF, these core targets were enriched in RNA polymerase II transcription factor activity (ligand), activated sequence-specific DNA binding, and hydrolysis activity (Figure 1C). The 153 genes were associated with 45 pathways (*p* < 0.05), among which the cancer, chemical carcinogenesis-receptor activation, ras signaling, and focal adhesion pathways were the most prevalent (Figure 1D).

### 3.3 Screening of core targets for overlapping genes and molecular docking

The 153 composite targets were input into the STRING database to remove the unconnected targets (combined score <0.7), and the PPI network was obtained. In total, 130 nodes and 405 edges were included in the network. According to the characteristics of network topology, the median values of DC, BC, and closeness centrality were 6.23, 357.78, and 0.17, respectively. Ten targets, MAPK1, SRC, MARK3, EGFR, ESR1, PTGS2, PTPN11, JAK2, PPKCA, and FYN, exceeded these values (BC and closeness centrality values higher than that of the median as well as a DC value twice that of the median) (Figure 1E). In detail, PTGS2, ESR1, MAPK1, PTPN11, and MAPK3 were potential anti-epileptic targets for linoleic acid; SRC and PRKCA for aloesone; FYN, EGFR, JAK2, PTGS2, and SRC for isoeleutherol glucosiden qt; and FYN, EGFR, JAK2, PRKCA, PTGS2, and SRC for anthranol (Table 2). SRC had the highest subgraph centrality value and was selected as the potential target for aloesone for further evaluation. To better understand the link between aloesone and the small G protein SRC, a molecular modeling study was carried out on the c-SRC crystallographic structure. Docking results demonstrated the hydrogen bonding interaction between the C2-oxopropyl of aloesone and the key Asp404 residue of c-SRC (Figure 1F), suggesting that c-SRC was the potential target for aloesone.

**TABLE 2:** The core targets of linoleic acid, aloesone, isoeleutherol glucosiden_qt, and anthranol.

Compounds	Core targets
Linoleic acid	PTGS2
	ESR1
	MAPK1
	PTPN11
	MAPK3
Aloesone	SRC
	PRKCA
Isoeleutherol glucosiden_qt	FYN
	EGFR
	JAK2
	PTGS2
	SRC
Anthranol	FYN
	EGFR
	JAK2
	PRKCA
	PTGS2
	SRC

### 3.4 Aloesone improved glutamate-induced injury of neuron cell by inducing phosphorylation of the SRC protein

Based on the results of GO analysis which revealed that 153 targets were highly enriched in neuron projection, closely associated with epilepsy (Srivastava et al., 2017; Doyle et al., 2021), we chose the mouse hippocampus neuron cell line (HT22) for further study. As the excessive release of glutamate leads to excitotoxicity and oxidative stress in neurons, contributing to the etiology of epilepsy, glutamate-treated HT22 cells are typically used to explore the effect of aloesone on glutamate-induced excitotoxicity (Chen S. et al., 2020; Chen X. et al., 2020).

**FIGURE 2:**
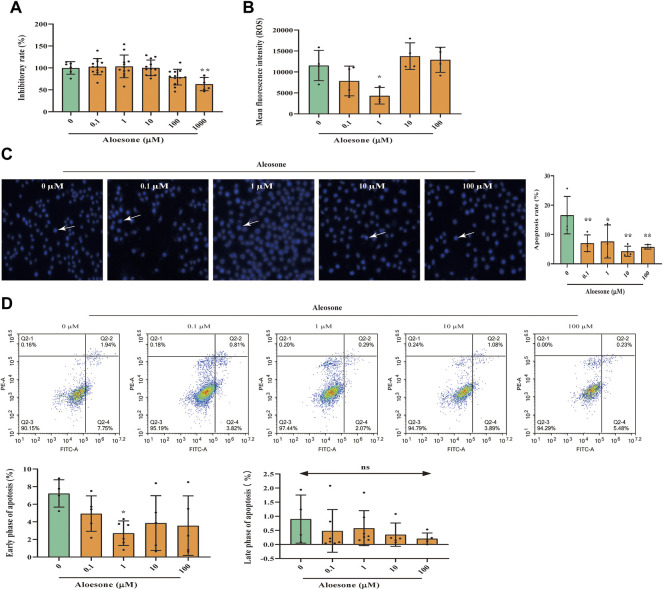
Effects of aloesone on glutamate-induced HT22 injury. **(A)** Inhibitory effect of aloesone (0.1–1,000 µM) on HT22 cells. **(B)** ROS content in HT22 cells treated with glutamate alone or a combination of glutamate and aloesone. **(C)** Representative images and analysis histograms of Hoechst staining. Cells with condensed chromatin and nuclear shrinkage underwent apoptosis. Apoptosis rate was calculated using the following formula: number of apoptosis cells/total cell. **(D)** Representative photographs for annexin Ⅴ-FITC and PI analyse. *p < 0.05 compared with the glutamate group.

First, we demonstrated that the administration of aloesone for 24 h did not affect the viability of HT22 cells, except at 1,000 µM (Figure 2A). Therefore, aloesone concentrations ranging from 0.1 to 100 µM were used in subsequent experiments.

The cells treated with 5 mM glutamate and 1 µM aloesone for 12 h showed decreased ROS production compared with those in the glutamate-alone group (Figure 2B). Results of Hoechst staining demonstrated that glutamate induced the condensation of chromatin and nuclear shrinkage. Compared with that of the glutamate alone group (16.6 ± 6.4), the total apoptosis rate of glutamate-treated cells with 0.1, 1, 10, and 100 µM aloesone significantly decreased (7.0 ± 2.8, 7.6 ± 5.6, 4.3 ± 1.7, 5.8 ± 0.8, respectively), compared to that in the glutamate alone group (Figure 2C). Further study, using a flow cytometer to analyze annexinⅤ–FITC, illustrated that 1 µM aloesone improved the early phase of apoptosis compared with glutamate alone group, but did not affect the late phase of apoptosis (Figure 2D).

**FIGURE 3:**
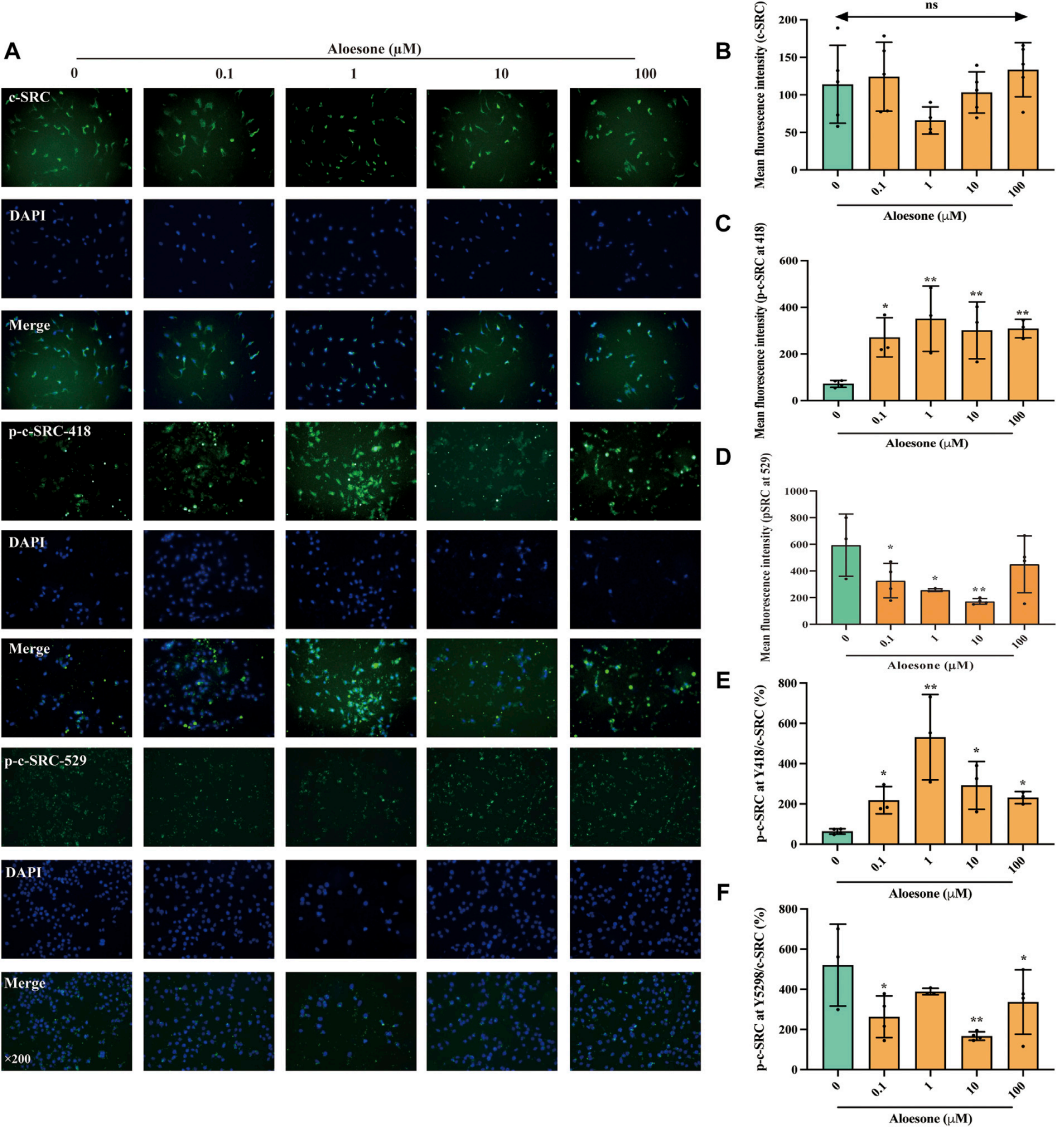
Immunofluorescence staining for c-SRC, p-c-SRC Y418, and p-c-SRC Y529 in HT22 cells treated with glutamate alone or combination of glutamate and aloesone. **(A)** Representative images. **(B–F)** Immunofluorescence density of c-SRC, p-c-SRC Y418, p-c-SRC Y529, ratio of p-c-SRC Y418 with c-SRC, and ratio of p-c-SRC Y529 with c-SRC. *p < 0.05 compared with the glutamate group.

As shown in our previous network analysis, SRC exhibited the highest subgraph centrality value, and Asp404 was likely the anchor site for aloesone. Hence, the expression and phosphorylation levels of c-SRC were determined using immunofluorescence. The c-SRC protein was located in the cytoplasm (Figure 3A), which is consistent with a previous study (Goh et al., 2010), whilein fluorescence density results illustrated that aloesone significantly increased the immunocontent of phosphorylated Y418 at c-SRC (Figures 3C,E) and decreased that of Y529 (Figures 3D,F), but did not influence the expression of c-SRC (Figure 3B).

### 3.5 Aloesone inhibited PTZ-induced acute and chronic seizures

**FIGURE 4:**
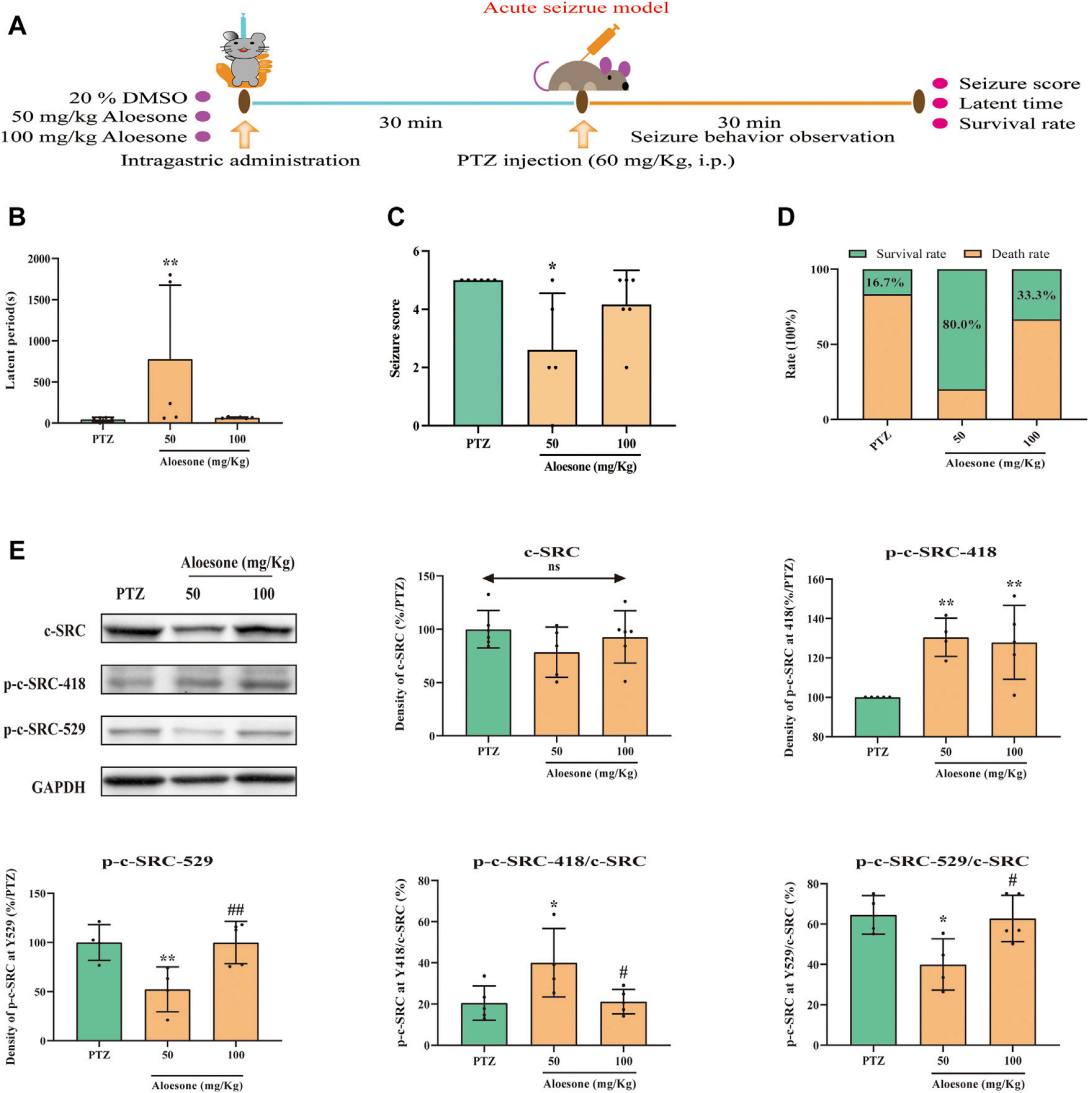
Effects of aloesone (50 and 100 mg/kg) on PTZ-induced acute seizures. **(A)** Procedure of inducing acute seizures with PTZ. **(B)** Latent period of PTZ-induced rats administered 20% DMSO only or aloesone (50 or 100 mg/kg). **(C)** Seizure score. **(D)** Survival and death rates. **(E)** Western blot images and lane density analysis of c-SRC, p-c-SRC Y418, p-c-SRC Y529, ratio of p-c-SRC Y418 with c-SRC, and ratio of p-c-SRC Y529 with c-SRC. *p < 0.05, **p < 0.01, compared with the PTZ group. #*p* < 0.05, #*p* < 0.01, compared with the 50 mg/kg aloesone group.

To confirm the effects of aloesone on seizures, PTZ, an inhibitor of the GABA receptor, was used to induce acute and chronic seizures. The latent period was significantly increased (777.60 ± 898.2 vs. 45.2 ± 26.7 s), and the seizure scores were reduced (2.6 ± 1.9 vs. 5.0 ± 0.0) in the 50 mg/kg of aloesone group compared with those in the PTZ-only group. Overall, the 50 mg/kg aloesone group demonstrated a markedly increased survival rate (80.0%), compared with rats in the PTZ group (16.7%) (Figures 4B–D). Furthermore, a higher level and decreased expression of phosphorylation at the Y418 and Y529 sites of c-SRC, respectively, were observed in the hippocampus of 50 mg/kg aloesone group compared with those in the PTZ group, suggesting that aloesone could inhibit seizures by increasing the activation of c-SRC (Figure 4E).

**FIGURE 5:**
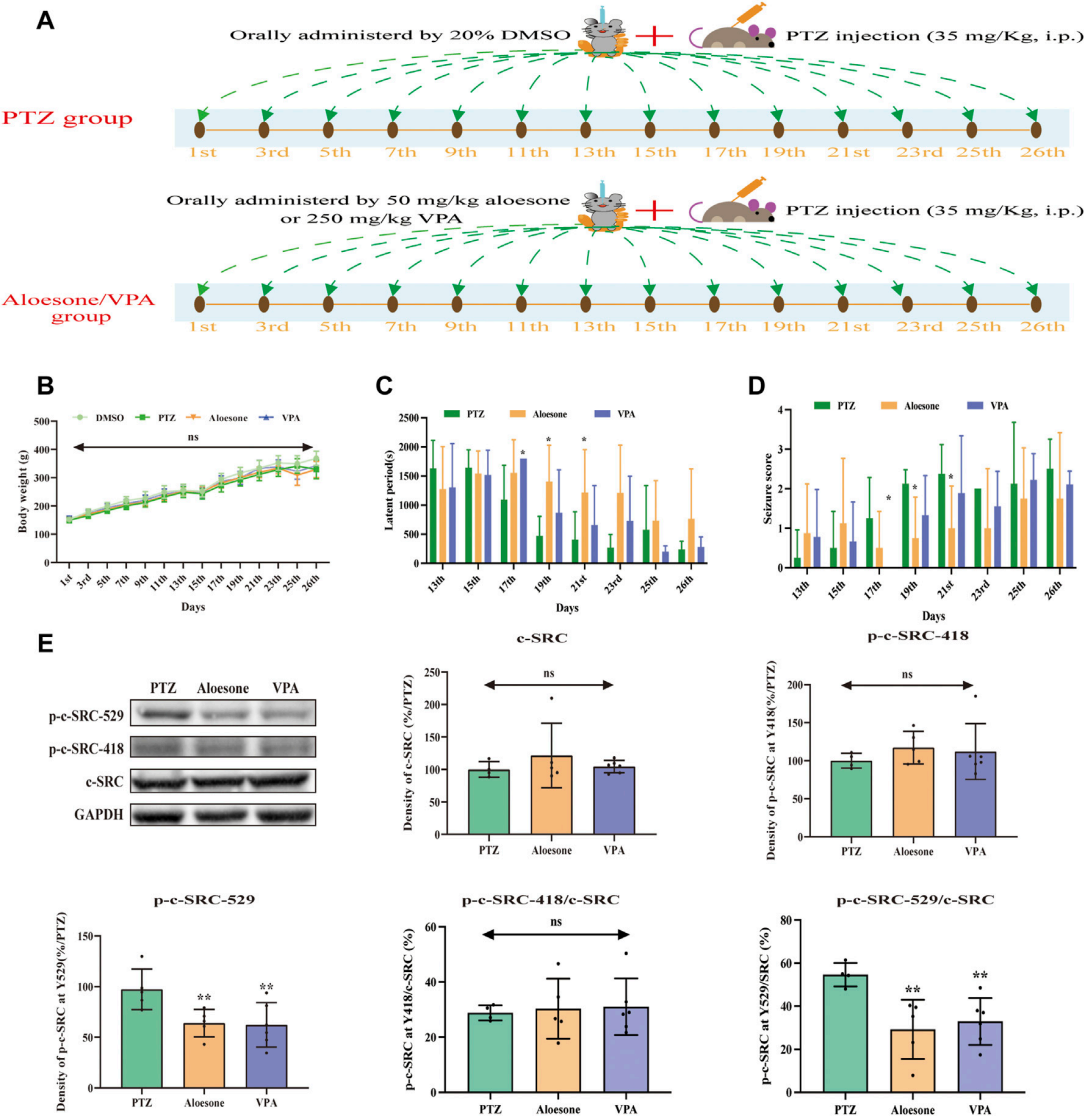
Effects of aloesone (50 mg/kg) on PTZ-induced chronic seizures. **(A)** Procedure of inducing chronic seizures with PTZ. **(B)** Body weights of rats administered 20% DMSO, PTZ, aloesone (50 mg/kg), or valproic acid (VPA, 350 mg/kg). **(C)** Latent periods in the PTZ, aloesone, and VPA groups. **(D)** Seizure score. **(E)** Western blot images and lane density analysis of c-SRC, p-c-SRC Y418, p-c-SRC Y529, ratio of p-c-SRC Y418 with c-SRC, and ratio of p-c-SRC Y529 with c-SRC. **p < 0.01 compared with the PTZ group. Graphical abstract. Aloesone from *Aloe vera* inhibits glutamate-induced HT22 injury and PTZ-induced seizures by increasing the activation of c-SRC, acting as a potential anti-seizure drug.

Chronic seizures were induced by injecting the rats with PTZ over a period of 26 days (Figure 5A). The results showed that 50 mg/kg aloesone did not affect the body weights of rats compared with those of the sham group at 26 days (Figure 5B). Most rats injected with PTZ (5/8) began experiencing seizures from day 17 (PTZ vs. DMSO group, respectively: seizure score: 1.2 ± 1.0 vs. 0 ± 0, latent period: 1,092.2 ± 591.4 vs. 1800 ± 0 s). At days 19 and 21, 50 mg/kg aloesone significantly controlled the seizures by reducing the seizure score and increasing the latent period compared with the PTZ group (seizure score: 0.75 ± 1.0 vs. 2.1 ± 0.3, latent period:1,551.5 ± 571.1 vs. 1,092.2 ± 591.4 s, Figures 5C,D). Furthermore, we confirmed the effect of aloesone on c-SRC by detecting the vital phosphorylation of c-SRC. Results demonstrated that the phosphorylation of c-SRC at Y529 was markedly reduced in the aloesone group compared with that in the PTZ-induced chronic seizure group (Figure 5E).

## 4 Discussion

The aim of this study was to explore the potential anti-seizure compounds in *Aloe vera* to promote its use. The network pharmacology of traditional Chinese medicine is a bioinformation network construction and topology analysis strategy based on high-throughput data analysis and virtual computing (Li and Zhang, 2013), that was generated to analyze traditional Chinese prescriptions, herbs, and their compounds. Using network pharmacology, we demonstrated that linoleic acid, aloesone, isoeleutherol glucosiden qt, and anthranol, which can potentially cross the BBB, may exert anti-seizure effects. Among these, linoleic acid has previously been reported to have anti-seizure effects. Specifically, Lauritzen et al. demonstrated that administration of 100 and 500 nM/kg linoleic acid protected 90% of CA1 and CA3 neurons against kainic acid (KA)-induced injury in rats (Lauritzen et al., 2000). Additionally, Ekici et al. reported that 100 mM/kg linoleic acid delayed the latent period by 1.8 times for PTZ-induced generalized tonic-clonic seizures and shortened the major seizure duration (7.5 ± 2.5 vs. 237.8 ± 104 s) (Ekici et al., 2014). In the present study, we demonstrated that PTGS2, ESR1, MAPK1, PTPN11, and MAPK3 may be the targets of linoleic acid against epilepsy. However, to our knowledge, no study has focused on the anti-seizure effects of aloesone, isoeleutherol glucose qt, and anthranol.

First, aloesone was selected as the target compound and its anti-seizure effect was determined. The imbalance of glutamatergic and GABAergic neurons is related to epileptogenesis, and glutamate is a vital neurotransmitter mediating seizure-associated cell death via oxidative stress or excitotoxicity (Coyle and Puttfarcken, 1993; Xie et al., 2021). Our results demonstrated that aloesone alleviated glutamate-induced neuronal injury by reducing the intracellular levels of ROS and inhibiting the early apoptosis of neurons. This finding is consistent with reports from previous studies that aloesone possesses radical scavenging activity (Lucini et al., 2015; Reza Nazifi et al., 2019). PTZ is a chemoconvulsant that induces seizures by inhibiting the GABA_A_ receptor and decreasing the calcium influx. A long-term subdose (20–35 mg/kg) and overdose (40–90 mg/kg) of PTZ can induce myoclonic jerks to generalized tonic-clonic seizures, mimicking absent epilepsy, as well as partial and generalized seizures in patients (Singh et al., 2021). Hence, these models are commonly used to explore new anti-seizure drugs. In the present study, 50 mg/kg aloesone significantly prolonged the latent period, inhibited the seizure score, and improved the survival rate of rats with PTZ-induced acute and chronic seizures, which suggested that aloesone may serve as a broad-spectrum anti-seizure drug that can block glutamate and activate GABA.

While the pathogenesis of seizures and epilepsy remains unclear, the discovery of new anti-seizure drugs that can delay the pathogenesis of seizure is vital. In this study, the non-receptor tyrosine protein (SRC), which is involved in the essential functions of neurons, including proliferation and apoptosis (Peng et al., 2019), was predicted to be the target of aloesone associated with epilepsy by network pharmacology and molecular docking. The SRC protein contains Src homology-2 (SH2), SH3, SH4, and a catalytic domain. Phosphorylation sites in SH2 (Y529) and SH4 (Y418) play opposite roles in the activation of SRC, of which the former inactivates the enzyme while the latter activates the enzyme. Moreover, previous studies have shown that c-SRC is closely associated with epilepsy. Reduced phosphorylation of c-SRC (Y418 and Y529) in the CA1 region has been reported in Li-Cl-induced epilepsy (Kim and Kang, 2021). However, in rats with KA-induced seizures, phosphorylation of Y418 significantly increases after the eighth day of administration of KA (Sharma et al., 2021). Activation of SRC has also been observed in models of PTZ-induced seizures (40 mg/kg in 24 h) (Putra et al., 2020). In this study, aloesone tended to increase and decrease the phosphorylation of c-SRC at Y418 and Y529, respectively, in glutamate-treated HT22 cells and PTZ-treated acute rats. This suggests that aloesone activates c-SRC but does not alter SRC expression. In rats with PTZ-induced acute seizures, 50 mg/kg of aloesone significantly activated c-SRC by simultaneously increasing and decreasing the phosphorylation levels at the Y418 and Y529 sites, respectively. However, 100 mg/kg aloesone significantly increased the phosphorylation at site (Y529) of c-SRC, which could suppress the activation of c-SRC. This phenomenon, wherein 100 mg/kg of aloesone simultaneously increased phosphorylation levels at the two opposite-function sites of c-SRC, may offset the increased activation of c-SRC, accounting for its inefficiency in seizures. In terms of PTZ-induced chronic seizures, 50 mg/kg aloesone activated c-SRC by reducing the phosphorylation of Y529.

This study has several limitations. First, although we confirmed the anti-seizure effects of aloesone, other potential compounds, such as isoeleutherol glucosiden qt and anthranol, were not evaluated due to the limitation of their synthesis. Second, as few studies have been conducted on the effects of aloesone in rats, we chose doses of 50 and 100 mg/kg aloesone for PTZ rats based on other compounds of *Aloe vera,* such as aloe-emodin, which exhibited similar anti-oxidative effects as aloesone in the brain (Lucini et al., 2015; Arcella et al., 2018; Xian et al., 2021; Pasala et al., 2022). However, concentrations below 50 mg/kg were not included in the present study. Hence, further studies should focus on the anti-seizure effects of low concentrations of aloesone. Finally, SRC was shown to be a potential target of aloesone; however, 3,068 protein targets were employed in SwissTargetPrediction which restricted the number of predicted targets for aloesone (Daina et al., 2019). Hence, other techniques, such as RNA-sequencing and proteomics, should be used to further explore the action mechanism of aloesone.

In conclusion, aloesone from *Aloe vera* was predicted to be a potential compound to treat seizures via network pharmacology. Both *in vitro* and *in vivo* experiments confirmed that aloesone inhibited the glutamate-induced HT22 injury and PTZ-induced seizures by increasing the activation of c-SRC. These findings indicate that aloesone may serve as a novel anti-seizure drug.

## Data Availability

The original contributions presented in the study are included in the article/supplementary material, further inquiries can be directed to the corresponding authors.
